# Optimized Polyethylene Glycolylated Polymer–Lipid Hybrid Nanoparticles as a Potential Breast Cancer Treatment

**DOI:** 10.3390/pharmaceutics12070666

**Published:** 2020-07-15

**Authors:** Salam Massadeh, Mustafa E Omer, Asmaa Alterawi, Rizwan Ali, Fayez H Alanazi, Fares Almutairi, Wejdan Almotairi, Faris F Alobaidi, Khulud Alhelal, Mansour S Almutairi, Abdulaziz Almalik, Aiman A. Obaidat, Manal Alaamery, Alaa Eldeen Yassin

**Affiliations:** 1 Developmental Medicine Department, King Abdullah International Medical Research Center, King Saud Bin Abdulaziz University for Health Sciences, King Abdulaziz Medical City, Ministry of National Guard-Health Affairs (MNG-HA), Riyadh 11481, Saudi Arabia; massadehsa@ngha.med.sa (S.M.); almutirima1@ngha.med.sa (M.S.A.); 2 KACST-BWH Centre of Excellence for Biomedicine, Joint Centers of Excellence Program, King Abdulaziz City for Science and Technology (KACST), Riyadh 11442, Saudi Arabia; aalmalik@kacst.edu.sa; 3 College of Pharmacy, King Saud bin Abdulaziz University for Health Sciences, Riyadh 11481, Saudi Arabia; ahmedm@ksau-hs.edu.sa (M.E.O.); terawia@ksau-hs.edu.sa (A.A.); alanazi4172@ksau-hs.edu.sa (F.H.A.); fares.motery@hotmail.com (F.A.); Almutairi041@ksau-hs.edu.sa (W.A.); Alobedy854@ksau-hs.edu.sa (F.F.A.); Alhilal081@ksau-hs.edu.sa (K.A.); obaidata@ksau-hs.edu.sa (A.A.O.); 4 King Abdullah International Medical Research Center (KAIMRC), Riyadh 11481, Saudi Arabia; 5 Medical Research Core Facility and Platforms, King Abdullah International Medical Research Center (KAIMRC), National Guard Health Affairs (NGHA), P.O. Box 22490, Riyadh 11426, Saudi Arabia; aliri@ngha.med.sa; 6 Life Sciences and Environment Research Institute, King Abdulaziz City for Science and Technology (KACST), Riyadh 11442, Saudi Arabia

**Keywords:** anastrozole, poly-caprolactone, PEGylated-polymeric nanoparticles, DSC

## Abstract

Purpose: The aim of this work is to optimize a polyethylene glycolated (PEGylated) polymer–lipid hybrid nanoparticulate system for the delivery of anastrozole (ANS) to enhance its biopharmaceutical attributes and overall efficacy. Methods: ANS loaded PEGylated polymer–lipid hybrid nanoparticles (PLNPs) were prepared by a direct emulsification solvent evaporation method. The physical incorporation of PEG was optimized using variable ratios. The produced particles were evaluated to discern their particle size and shape, zeta-potential, entrapment efficiency, and physical stability. The drug-release profiles were studied, and the kinetic model was analyzed. The anticancer activity of the ANS PLNPs on estrogen-positive breast cancer cell lines was determined using flow cytometry. Results: The prepared ANS-PLNPs showed particle sizes in the range of 193.6 ± 2.9 to 218.2 ± 1.9 nm, with good particle size uniformity (i.e., poly-dispersity index of around 0.1). Furthermore, they exhibited relatively low zeta-potential values ranging from −0.50 ± 0.52 to 6.01 ± 4.74. The transmission electron microscopy images showed spherical shape of ANS-PLNPs and the compliance with the sizes were revealed by light scattering. The differential scanning calorimetry DSC patterns of the ANS PLNPs revealed a disappearance of the characteristic sharp melting peak of pure ANS, supporting the incorporation of the drug into the polymeric matrices of the nanoparticles. Flow cytometry showed the apoptosis of MCF-7 cell lines in the presence of ANS-PLNPs. Conclusion: PEGylated polymeric nanoparticles presented a stable encapsulated system with which to incorporate an anticancer drug (ANS) with a high percentage of entrapment efficiency (around 80%), good size uniformity, and induction of apoptosis in MCF-7 cells.

## 1. Introduction

Breast cancer is considered to be the second leading cause of cancer-related death both worldwide and in Saudi Arabia [1]. Since the discovery of the dependence of breast cancer cells on estrogen and the presence of estrogen receptors in the cell walls of many species of cancer cells, the mode of treatment has been revolutionized by the long-term use of antiestrogen drugs, in addition to chemotherapy or radiotherapy [2]. Tamoxifen, the first drug approved for estrogen positive breast cancer treatment, has resulted in a tremendous increase in the survival rate among breast cancer patients [3]. However, despite the prominent advantages of tamoxifen, the risk of developing uterine cancer and thromboembolic events are considered major limitations, alongside several side effects such as the alteration of menstruation [4,5,6].

Anastrazole (ANS), a third-generation aromatase inhibitor, has been proven to be superior to tamoxifen for reducing the recurrence of invasive breast cancer in postmenopausal women who are hormone-receptor positive [7]. Anastrazole was also recommended as a rational treatment option for hormone-receptor positive postmenopausal women with ductal carcinoma in situ [8]. It demonstrated significant reduction in the most serious adverse effects of tamoxifen, i.e., ischaemic cerebrovascular disorders and development of endometrial cancer [9]. The recommended regime is 1 mg tablet per day for 31 months [10]. The poor aqueous solubility of ANS results in variation of intestinal absorption and highly variable blood levels leading to undesirable side effect including thrombocytosis, osteoporosis, and vaginal bleeding [11].

Nanodrug delivery is an effective tool that massively contributes to optimize the outcomes of cancer therapy [12]. Nanoparticles are uniquely shaped via their physical and chemical properties [13]; their unique properties allow the incorporation of anticancer drugs in nanoparticle delivery systems (NPDS). NPDS overcome the barriers encountered with conventional treatments. Furthermore, NPDS have many advantages, such as, increased surface-to-volume ratio which leads to high activity, the ability to freely diffuse in the biological system very close to the cell membrane, and the capability to localize in the cancer tissue mostly due to enhanced the permeation and retention (EPR) phenomena which is a passive retention mechanism in a tumor, resulting in the accumulation of conventional drug delivery with prolonged circulation time [14,15,16], accomplished by the PEGylation delivery system, and controlled or sustained release of the drug [17].

Moreover, NPDS improve the biodistribution, which is a crucial element necessary to succeed in cancer therapy and maximize the therapeutic index. The co-delivery of P-gp inhibitors along with anticancer drugs in nanoparticle systems is a useful approach to overcome MDR [18]. Moreover, polyethylene glycolated (PEGylated) nanoparticles, as stealth nanoparticles, have been proven efficient in prolonging the circulation and decreasing the influence of RES uptake on the treatment [19]. These stealth nanoparticles reduce the degree of opsonization via a steric hindrance or by increasing the hydrophilicity of the surface [20,21,22]. Physical PEGylation has several advantages over chemical attachment of PEG on the surface of the NPs including, in addition to simplicity, rapidness, and low cost, the absence of premature drug release during the conjugation step [23].

Furthermore, a polymeric nanoparticle drug delivery system is usually a matrix system encompassing a drug in a natural or synthetic polymer [24]. Biodegradable polymers such as polylactic acids, polyglycolic acid, or their copolymer hybrids are extensively used due to their prolonged biodegradation rates and safe biological fate [25]. Polycaprolactone (PCL) is a biodegradable polymer with unique physical, mechanical, and rheological properties [26,27]. It shows eminent bioresorbable and biocompatibility properties [28,29] which permit a wide range of biomedical applications including implantation devices and nanodrug delivery systems [28,29,30]. PCL was successfully used as a nanodrug carrier for many anticancer drugs such as 5-flourouracil, paclitaxel, docetaxel, methotrexate, and doxorubicin [30,31,32,33,34].

Polymer–lipid hybrid nanoparticles (PLNPs) have recently emerged as a new promising nanoparticulate drug delivery system option through the combination of the main advantages of both lipid nanoparticles, including higher cellular penetrability and biological compatibility and polymeric nanoparticles beneficial features such as enhanced biostability and drug release prolongation ability [12,13,35,36]. PLN formulations have been known to induce tremendous enhancement in the efficacy of doxorubicin and mitomycin C through the inhibition of breast cancer resistance protein (BCRP+) and multidrug resistance protein 1 (MRP1+), which are overexpressed in many human breast cancer cells [17,37,38].

In this study, we encapsulated ANS in PLNPs to improve its solubility and to lower the incidence of side effects. Furthermore, we evaluated the apoptotic response of the ANS-PLNPs in breast cancer cell lines.

## 2. Materials and Methods

### 2.1. Materials

Anastrozole (ANS) (molecular weight 293.37, purity ≥ 98% (HPLC), polyvinyl alcohol (PVA), polycaprolactone (PCL, Mw 42,000 Da), stearic acid (SA), and PEG 6000 were purchased from Sigma-Aldrich Chemical Co. (St. Louis, MO, USA). The breast cancer cell line MCF-7 (ATCC HTB-22) was purchased from the American Type Culture Collection (Manassas, VA, USA). The cells were cultured in Dulbecco’s modified Eagle medium DMEM media from Gibco Laboratories (Gaithersburg, MD, USA) containing 10% fetal bovine serum and 1% L-glutamine cells and were incubated at 37 °C in a 5% CO_2_ humidified incubator. An Annexin V-FITC apoptosis detection kit (BMS500FI-100, was purchased from Invitrogen, Carlsbad, CA, USA). Cells were harvested using the appropriate amount of trypsin Triple Express 1× from (Gibco Laboratories, Gaithersburg, MD, USA). All other reagents and chemicals were of analytical grade.

### 2.2. Identification of ANS Pure Drug

ANS was subjected to FT-IR spectrophotometry (Cary 630; Agilent Technologies, Santa Clara, CA, USA) for qualitative identification. Then, the spectrum was analyzed in order to compare it with standard IR spectrums of ANS reported in the literature.

### 2.3. Preparation of ANS Polymeric Nanoparticles

The calculated quantity (10 mg) of ANS was weighed accurately and dissolved in 20 mL of distilled water using an ultrasonic bath at 35 °C to formulate a 0.5 mg/mL ANS solution. In a separate container, 20 mg of polycaprolactone with 5 mg of stearic acid were dissolved in 10 mL of chloroform. Then, in a beaker, 2 mL of solution A were mixed with 10 mL of solution B. To create the emulsion, the two solutions were probe sonicated (Q700; Qsonica LLC, Newton, CA, USA) at 40% intensity for three minutes under an ice bath. Finally, the formed emulsion (12 mL) was dispersed in 15 mL of 2% polyvinylalcohol (PVA) and another 3 min probe-sonication cycle was made under ice. The final formed double emulsion was stirred for three hours at room temperature under a fume hood to facilitate the complete evaporation of chloroform. The formed nanosuspension was centrifuged (Heraeus Megafuge 16R; Thermo Fisher Scientific, Waltham, MA, USA) in Eppendorf tubes at 25,200 rcf for 30 min at 4 °C. Then, the supernatant was withdrawn and collected in a separate container. The precipitated nanoparticles were washed with distilled water and kept in a −30 °C freezer. After 24 h, the nanoparticles were dried by freeze-dryer (BETA 2–8 LDplus; Martin Christ Gefriertrocknungsanlagen GmbH, Osterode am Harz, Germany). Table 1 lists the four formulations prepared.

### 2.4. Drug Analysis by Ultraviolet (UV) Spectrophotometry Method

In order to measure the content uniformity and percentage entrapment efficiency (EE%) of ANS, ANS solutions with different concentrations were prepared and their UV absorbance was determined at λ = 263 nm using a UV spectrophotometer (Evolution 60 S; Thermo Fisher Scientific, Waltham, MA, USA). Then, the absorbances of all standard solutions were plotted versus their concentrations to create an ANS calibration curve. 

### 2.5. Evaluation of the Prepared ANS Nanoparticle Formulations

#### 2.5.1. Measurement of Particle Size and Polydispersity Index

A sample from each freeze-dried ANS nanoparticle formulation was taken and dispersed in distilled water (0.1% *w*/*v*) in a bath sonicater. Using a particle size analyzer (ZetaPALS; Brookhaven Instruments, Holtsville, NY, USA) with an angle of detection of 90°, the particle size and polydispersity index were measured and calculated as averages of three readings.

#### 2.5.2. Measurement of Zeta-Potential

Using the particle size analyzer (ZetaPALS; Brookhaven Instruments, Holtsville, NY, USA), the zeta-potential was measured for all formulations (T1, P1, P2, and P3) by applying the laser Doppler velocimetry (LDV) mode.

#### 2.5.3. Particles Morphology

Transmission electron microscope (TEM) measurements were performed using a (JEM-1400 electron microscope; JEOL, Tokyo, Japan) operating at an acceleration voltage of 120 kV. A drop of the sample (1 mg/mL) was placed on a 400-mesh carbon-coated copper grid. The samples were air dried at room temperature prior to measurement.

#### 2.5.4. Measurement of Drug Entrapment Efficiency and Drug Loading

The supernatants, which were collected after the centrifugation processes (as mentioned previously in the preparation of the ANS polymeric nanoparticles) for each formulation, were filtered using a syringe filter (Whatman 0.2 µm PTFE). Then, the concentration of ANS was measured using the abovementioned UV spectrophotometry method. Finally, the percentage of drug entrapment efficiency (%EE) was calculated according to the following equation: %EE = (weight of entrapped drug)/(weight of initial drug) × 100.

### 2.6. ANS Release Study

The percentage of ANS released from each NP formulation was determined by incorporating 1 mL of NP dispersion in phosphate buffer (pH 7), containing an amount equivalent to 1 mg ANS, inside a dialysis tube (cutoff size 12,000 Da) firmly tied from one end. After tying the other end, the tube was immersed in a vessel containing 20 mL of the same media and placed in a shaking water bath adjusted to 37 °C ± 1 °C and 80 rpm. Samples of 1 mL were withdrawn at predetermined time intervals and replaced by fresh preheated medium to maintain the sink condition. The percentage of ANS released was determined in each sample using the same spectrophotometric method.

### 2.7. Evaluation of the Anticancer Activity Using Flow Cytometry

A eBioscience Annexin V-FITC apoptosis detection kit was used to evaluate cell viability as per the manufacturer’s recommendation. Briefly, MCF-7 cells at passage number 12 were seeded (0.4 million) in a T25 culture flask in 3 ml volume of 10% FBS complete Dulbecco’s modified Eagle medium (DMEM) from Gibco. After 24 h incubation, cells were treated with 0.03 μM ANS-PLNPs (PCL-Stearic acid), PEGylated ANS-PLNPs (PCL-Stearic acid-PEG), Void NPs, 0.015 μM ANS, and 0.030 μM ANS. In addition, two T25 flasks for controls with binding buffer and annexin-V. After treatment for 48 h, supernatant and attached cells were collected by centrifugation. The collected cells were washed twice with PBS, then centrifuged (600× *g*, 5 min, RT). Cell-viability was measured using 5 μL Annexin V-FITC added to cell suspension, cells were incubated for 10 min at room temperature, then, washed with binding buffer. A total of 10 μL propidium iodide (20 μg/mL) were added to the cells’ suspension. FACS analysis were performed by using FACS Canto II Flow cytometer system (BD Biosciences, San Jose, CA, USA).

### 2.8. Fluorescence High Content Imaging

MCF-7 cells were plated in 96-well plates at a density of 5000 cells per well. Cells were treated with anastrozole (0.03 μM and 0.3 μM), anastrozole loaded T1 and P3 NPs (0.03 μM and 0.3 μM), and void NPs for 0, 24, 48, and 72 h. After treatment, cells were stained with calcein AM (2 μg/mL), HOECHST33342 (5 μg/mL), and propidium iodide (2.5 μg/mL) for 20 min at 37 °C and 5% CO_2_. Cells were, then, imaged using a Molecular Devices ImageXpress^®^ Micro and analyzed using MetaXpress^®^ software, Molecular Devices, Downingtown, PA, USA. Nuclei were counted from each well, and average fluorescence intensity was calculated, covering approximately 60% of a single well of 96-well plates. All experiments were performed in triplicates, averaged, and values were reported as mean ± SD.

## 3. Results

**Figure 1:**
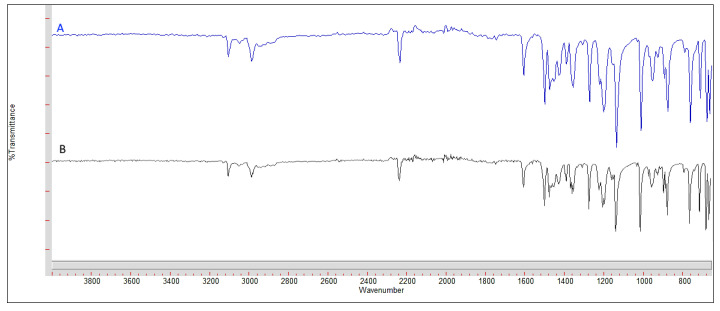
FT–IR spectrum of used ANS and standard ANS. Spectrum (**A**) represents the standard ANS spectrum and (**B**) represents the used ANS.

To identify the model drug (ANS), Figure 1 compares the FT–IR spectrum of the ANS used in our experiments with the reference standard in the literature [39]. The fingerprint IR spectrum regions for both compounds were identical, which demonstrates that the used ANS is pure and free from any impurities.

The results of the mean particle size, polydispersity index, zeta-potential, and EE% for all the prepared ANS-PLNPs formulations are summarized in Table 2. The values of the mean particle sizes ranged from 193 to 218 nm. Overall, there was no significant difference in particle size between the three PEGylated formulations (P1, P2, and P3). However, a significant decrease in the size was detected between T1 and P3, having the same composition except for the incorporation of PEG 6000 in P3. This can be attributed to the decrease in the internal contraction force of the polymeric nanoparticle network induced by PEG 6000 leading to a slight increase in particle size. As the PEGylated formulations exhibit a close range of particle size, this indicates that the PEG/PCL ratio had no effect on particle size.

All the formulations showed low values of polydispersity index (around 0.1). It is agreed upon that polydispersity values below 0.7 indicate monodispersed property of the prepared NPs. This result confirms that the variation in the composition (formulation variables) did not impact the polydispersity index of ANS-PLNPs.

The zeta-potential values of all ANS-PLNPs formulations are presented in Table 2. Generally, 30 mV is considered to be a critical zeta-potential value for the stability of nanoparticles [33]. Our results revealed low zeta-potential values of less than 5 mV, indicating the need for electrolyte adjustment when stored as dispersion. All PEGylated formulations had +ve zeta-potential values, while the non-PEGylated formulations had −ve values. As a formulation variable, PEG content has a significant effect on zeta-potential; this is the result of the high molecular weights of PEG used (6000), which have high positive charges on the polymer surface. This phenomenon was confirmed in a previous study conducted by Luangtana et al. [40].

The encapsulation efficiency (EE) for ANS-PLNPs are presented in Table 2, where a mean %EE of around 80% is noted. This supports the robustness of the preparation method in loading a high percentage of ANS. This finding (%EE) is close to a previously reported result (85.7%) for polymer–lipid nanoparticles reported by Dong et al. [41]. Referring to Table 2, it is very clear that the formulation variables PEG content (T1 vs. P1, P2, and P3) and ANS/PCL ratio (P1 vs. P2 vs. P3) have no significant effect on %EE.

**Figure 2:**
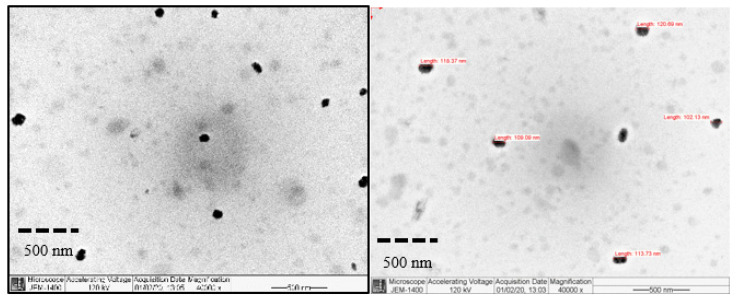
Transmission electron microscopy micrographs of the PLNP (P2) composed of 7.5 (PEG):30 (PCL):5 (SA):1 (ANS).

Figure 2 represents the TEM images of P2 formulation as a representative to PLNPs formulation. It is noticed that the particle sizes were lower than those determined by DLS which is very common in the literature as a result of particle swelling and the inclusion of the stagnant solvent layer in the measurement with DLS [38]. The images reveal almost spherical shapes with some irregularity that can be attributed to the presence of PEG molecules on the surface.

**Figure 3:**
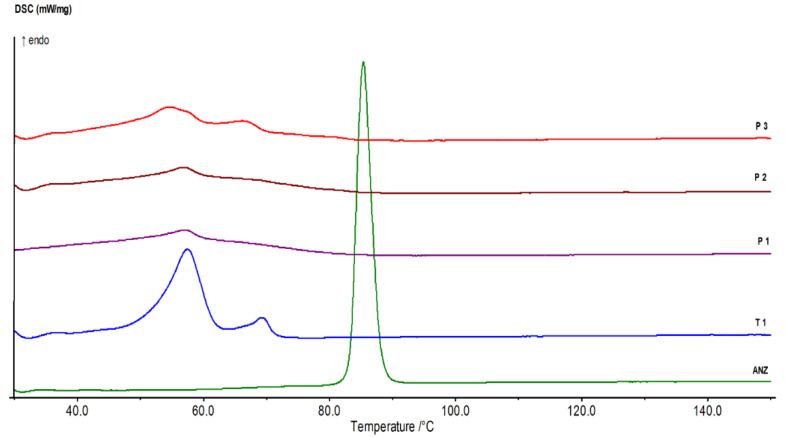
DSC thermograms of ANS and ANS-PLNPs formulations.

The thermal profile of the AN-PLNPs formulations are shown in Figure 3. The pure ANS demonstrated a sharp endothermic peak at 84.7 °C, which is related to the ANS melting point that was very close to a previously indicated DSC pattern [36] that revealed the DSC peak for pure ANS at 83.8 °C. The disappearance of a high sharp peak of ANS-PLNPs indicates the efficiency of this preparation method in the encapsulation of ANS inside a polymeric-nanoparticle system. The T1 thermogram shows two prominent endothermic peaks; the first at 57.4 °C and the second at 68.4 °C corresponding to the melting of PCL and stearic acid, respectively. The thermograms of P1, P2, and P3 depict the broadness of both peaks due to the inclusion of PEG 6000 in their composition which is known to melt in the same range [37,38,42,43,44].

**Figure 4:**
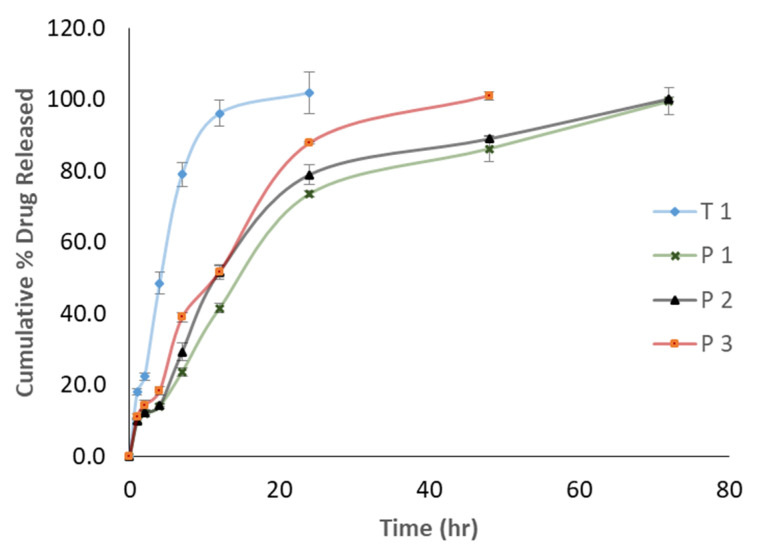
In vitro anstrazole release profiles from different PLNPs formulations.

Figure 4 depicts the ANS release profiles from the four PLNPs formulations. Specifically, the release profile appears different than the common biphasic pattern exhibited by most polymeric and solid lipid nanoparticles. All ANS-PLNPs showed no burst effect in their release profile.

The rate of ANS release was different in the four formulations. T1 had the fastest rate among the four formulations, while P1 had the slowest rate. After 12 h, cumulative % released values of 96%, 41%, 51%, and 51% were recorded for T1, P1, P2, and P3, respectively. Both P1 and P2 formulations showed a similar release profile extending for 72 h, with a slightly slower period after 24 h. A comparison of the ANS release profile from P3 with the T1 formulation facilitates an interesting conclusion that PEGylation can delay drug release from the PLNPs.

**Figure 5:**
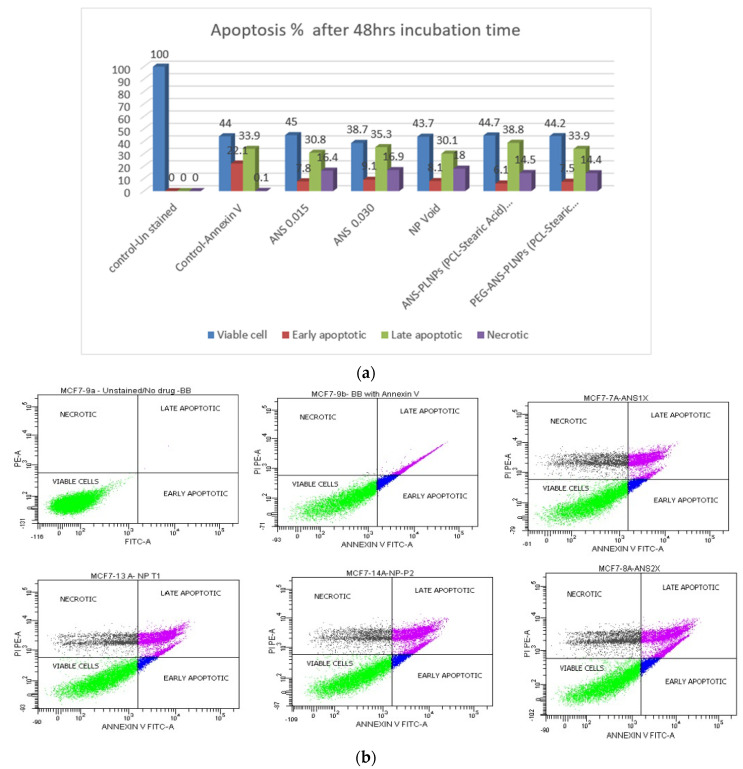
Flow cytometry results for MCF-7 cell lines incubated with ANS-PLNPs for 48 h. (**a**) A column graph representing the percentage of early apoptotic cells, late apoptotic cells, and necrotic cells. (**b**) Flow cytometry charts for MCF-7 cells.

FACS has been used to assess the apoptotic effect of the ANS-PLNPs on estrogen positive breast cancer cell lines. The analysis showed the percentages of early apoptotic, late apoptotic, and necrotic MCF-7 cells after 48 h of incubation with ANS free drug and ANS-PLNPs, Figure 5.

Regarding the ANS-PLNPs treatments, for both conditions (PCL and stearic acid) and (PCL, stearic acid, and PEG), at the same concentration of 0.03 µM, a very similar percentage for all parameters among early apoptotic, late apoptotic, and necrotic cells was noted.

## 4. Discussion

The incorporation of ANS into PEGylated PLNPs was meant to overcome the low dissolution and variable absorption rates of ANS and to prolong the residence time inside the body. PCL was selected as a drug scaffold based on numerous success stories with a variety of anticancer drugs [31,34,37]. The higher flexibility of the polymer chain allowed for producing low particle size NPs. It has been reported that PCL was superior to PLGA in producing lower particle size NPs [34,42].

The adopted method of preparation provided a simple, single step, reproducible technique for producing a physically PEGylated PLNPs with attractive attributes of uniform low particle sizes and high drug entrapment affinity. The reproducible production of low particle size and polydispersity indices proves the superior NPs physical stability and the appropriateness of the preparation procedure. PVA, a polymeric surfactant with hydrophilic nature, was commonly used to stabilize the primary emulsion in the double emulsion method for the synthesis of polymeric nanoparticles [43,44]. It has been proven to grant a mucoadhesive property to the surface of NPs, and thus has been exploited for many mucosal applications in vaginal drug delivery [42,43]. This would be of great impact on orally administered NPs. The unusual low zeta-potential values obtained with non-PEGylated PLNP formulation (T1) is highly attributed to the neutralizing effect induced by the high concentration (2%) of PVA solution used [44]. The 6000 Da of PEG was selected to provide optimum shielding to the NPs since a minimum of 5000 Da has been found crucial [44].

The broadness of the DSC crystalline melting peak of PCL in the thermograms of the PEGylated formulations (P1, P2, and P3) is considered an indication of more flexibility of the PCL chain and the deeper incorporation of ANS inside the particle matrix.

The formulation composition was designed to explore the impact and extent of PEGylation and drug to polymer ratio on the PLNPs attributes, including the prolongation of drug release. It has been shown that the drug to polymer ratio did not impact the physical characteristics of the PLNPs but slowed the ANS release rate. This agrees with what was reported by Ashour et al. [34]. They showed that PEGylation accelerated the rate of release of 5-FU. The PEGylation has clearly resulted in reduction in NPs sizes and significant prolongation of the ANS rate of release. This wa unlike what was reported by Machado Cruz et al. [45] that no impact on the particle size of itraconazole nanoparticles was caused by PEGylation. The ANS release prolongation effect can be attributed to their ability to induce crystalline imperfection in PCL allowing for deeper entrapment of ANS inside the particle matrix. This was confirmed from the DSC thermograms of PEGylated PLNPs. The absence of any ANS burst release effect can be ascribed by the use of high drug to polymer ratios (1:20, 30, and 40) and the well crystalline nature and slow hydration rate of PCL [45]. The hydrophobic nature of ANS can explain the slower rate of release observed with P3 (PEGylated) as compared with that obtained with T1 (non-PEGylated) as it exhibits delay crossing the PEG 6000 surface layer. This effect is reversed for hydrophilic drugs such as 5-FU [34].

The MCF-7 cells incubated with ANS start showing late apoptotic and necrosis of the cells as compared with the controls indicating an apoptotic effect of the aromatase inhibitor, ANS. Likewise, it is noticed that the ANS-PLNPs show a similar apoptotic profile of the MCF-7 cells as the free form of the drug. In conclusion, the ANS loaded PLNPs and the free form both induced late apoptosis and necrosis as compared with the control samples. Our results comply with previously published reports [37], which showed that ANS induced late apoptosis and necrosis of MCF-7 cells after 48 h of incubation time.

In addition to the FACS analysis, more studies including nanoparticle uptake profiles ans cell signaling pathways would provide more insight. Furthermore, transgenic or tumor-bearing animal models could provide more data about the therapeutic efficacy of this system.

**Figure 6:**
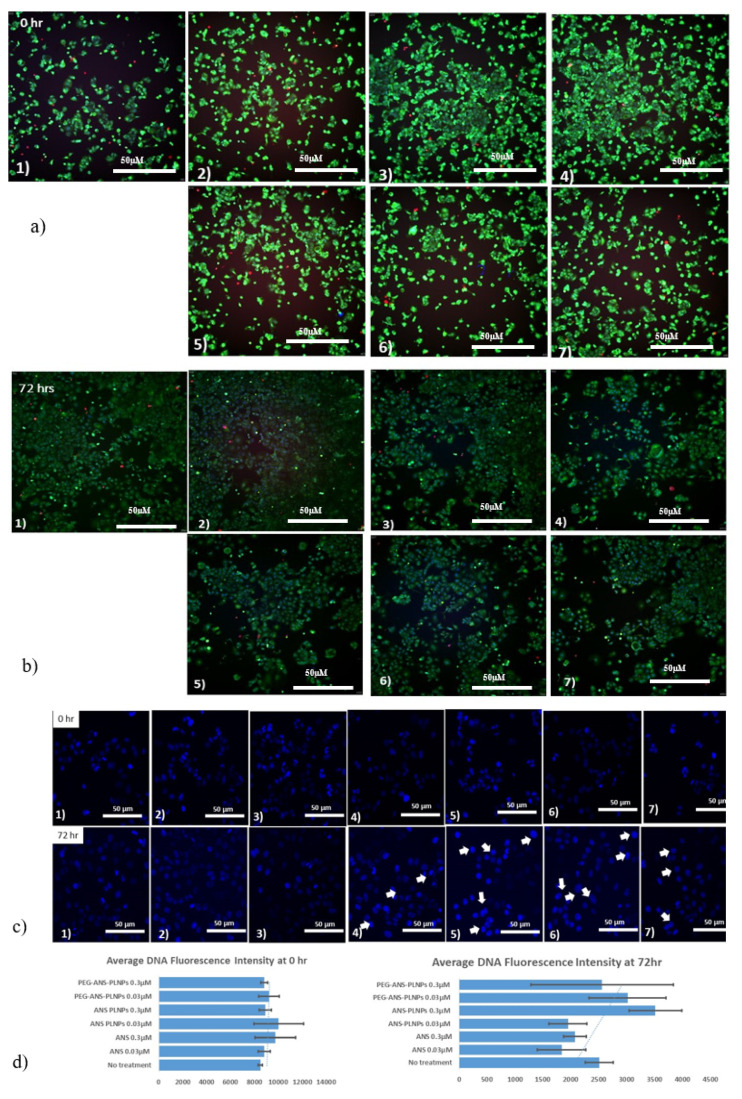
Fluorescence high content imaging of MCF-7 cells. MCF-7 cells were treated with (1) vehicle, (2) 0.03 µM anastrozole, (3) 0.3 µM anastrozole, (4) 0.03 µM PEG-ANS-PLNPs, (5) 0.3 µM PEG-ANS-PLNPs, (6) 0.03 µM ANS-PLNPs, and (7) 0.3 µM ANS-PLNPs for 0 h and 72 h. After treatment, cells were stained with calcein AM (2 μg/mL), HOECHST33342 (5 μg/mL), and propidium iodide (2.5 μg/mL) for 20 min. (**a**,**b**) Represent the merged images at times 0 and 72 h; (**c**) Represents the cells stained with HOECHST 33342; and (**d**) Represents the bar graphs of average nuclear fluorescence intensities of the MCF-7 cells at times 0 and 72 h. Scale bar of all images is 50 µm.

Furthermore, nuclear condensation and fragmentations are the classical hallmarks of apoptosis. It is well known that condensation of the nucleus occurs during the early stages of apoptosis. Blue fluorescent Hoechst 33342 brightly stains the condensed [46,47] chromatin of apoptotic cells, increasing the fluorescent intensity of the nucleus, whereas the normal chromatin of live cells is dimly stained. Hoechst 33342/Calcenin/PI staining was done to identify the necrotic cells by high content fluorescence imaging. Figure 6a,b shows the change in cellular behavior and morphology in the treated MCF-7 cells as compared with the untreated cells. In addition, the nuclei of the apoptotic cells gave a brilliant blue color (Figure 6c,d), which indicates DNA malformation of the cells that eventually lead to the inhibition of cellular proliferation. Figure 6c shows an increase in the nuclear fluorescence intensity after 72 h of cells treated with ANS-PLNPs and PEG-ANS-PLNPs as compared with the intensity at time 0. This points towards the presence of a significant population of DNA-damage related cell death due to the release of the ANS from the PLNP [48,49].

The encapsulation of ANS in PLNPs was presented here, for the first time, with a rationally selected composition to impart in improving the biopharmaceutical behavior toward significant enhancement in the therapeutic efficacy of the drug. The prepared ANS-PLNPs have many attractive features for per-oral delivery including the drug release duration and uniformity, the potential mucoadhesive properties induced by PVA, and the possible lymphatic uptake triggered by stearic acid. Thus, our PLNPs system can overcome the incomplete absorption resulting from the poor solubility of ANS and the achieved prolonged release can extend the drug residence time in the body leading to possible reduction of the dose, and consequently, all related adverse effects. In addition, the apoptotic effect induced by the particles is an interesting observation that adds another mode of activity enhancement for the treatment of breast cancer in postmenopausal women.

## 5. Conclusions

In this study, ANS loaded PLNPs were successfully optimized to low particle sizes (below 200 nm), low polydispersity indices (≈0.1), high EE% (≈80%), and high stability. In addition, PLNPs showed apoptotic effect on the estrogen positive breast cancer cell line as compared with the free form of the drug. In conclusion, the prepared PLNPs have high potential to enhance the therapeutic performance of ANS that make them worthwhile for further in vivo evaluation.

## Figures and Tables

**Table 1:** The composition of prepared anastrozole polymer–lipid hybrid nanoparticles (ANS-PLNPs) formulations.

Formulation	PEG 6000 (mg)	PCL (mg)	SA (mg)	ANS (mg)
T1	-	20	5	1
P1	10	40	5	1
P2	7.5	30	5	1
P3	5	20	5	1

**Table 2:** Mean particle size, polydispersity index, zeta-potential, and %EE efficiency of ANS-PLNPs formulations.

Formulation ID	Mean Particle Size (nm)	Polydispersity Index	Zeta-Potential	%EE
T1	218.16 ± 1.91	0.11 ± 0.02	−0.50 ± 0.52	79.7
P1	202.01 ± 2.02	0.10 ± 0.02	2.56 ± 6.78	80.1
P2	205.96 ± 4.04	0.11 ± 0.02	1.69 ± 3.68	80.4
P3	193.60 ± 2.89	0.12 ± 0.01	6.01 ± 4.74	81.3

All results are presented as an average of three measurements ± standard deviation.
